# Comparative analysis of microbiota in the ceca of broiler chickens with necrotic enteritis fed a commercial corn diet or with corn high in flavonoids (PennHFD1)

**DOI:** 10.3389/frmbi.2023.1212130

**Published:** 2023-10-25

**Authors:** Katarzyna B. Miska, Monika Proszkowiec-Weglarz, Vinicius Buiatte, Mahmoud Mahmoud, Tyler Lesko, Mark C. Jenkins, Surinder Chopra, Alberto Gino Lorenzoni

**Affiliations:** ^1^ Animal Biosciences and Biotechnology Laboratory, USDA-ARS, NEA, Beltsville Agricultural Research Center, Beltsville, MD, United States; ^2^ Department of Animal Science, College of Agricultural Sciences, The Pennsylvania State University, University Park, PA, United States; ^3^ Department of Plant Sciences, College of Agricultural Sciences, The Pennsylvania State University, University Park, PA, United States; ^4^ Animal Parasitic Diseases Laboratory, USDA-ARS, NEA, Beltsville Agricultural Research Center, Beltsville, MD, United States

**Keywords:** coccidia, ceca, Clostridium perfringens, Eimeria maxima, 16S

## Abstract

Necrotic enteritis (NE) is a disease of the gastrointestinal tract that is common in broiler chickens and causes substantial economic losses to the poultry industry worldwide. The removal of many antimicrobials in poultry diets has driven the search for alternatives. The purpose of this study was to determine the microbiota changes in the cecal luminal (CE-L) and mucosal (CE-M) populations of broiler chickens undergoing clinical NE (co-infected with *Eimeria maxima* and *Clostridium perfringens)* while fed a diet containing a flavonoid rich corn (PennHFD1) or control diet using commercial corns. It was previously shown that chickens fed a diet high in flavonoids had improved performance parameters, lower mortality rate, and lower incidence of intestinal lesions. Flavonoids have been shown to have anti-bacterial, immuno-modulatory, and anti-inflammatory activity. The current study included four experimental groups: infected chickens fed commercial corn diet (CTRL-A) or PennHFD1 (CTRL-B) and infected chickens fed commercial corn diet (IF-A) or PennHFD1 (IF-B). We found that most of the microbiota changes were due to infection rather than diet. The alpha diversity in the IF chickens was lower in both CE-L and CE-M. The beta diversity of microbial communities was different between IF and CTRL chickens, as well as between CTRL-A and CTRL-B. The beta diversity of CTRL birds was more homogenous compared to IF samples. Taxonomic analysis showed a decrease in short chain fatty acid producing bacteria in IF birds. An increase in lactic acid producing bacteria, *Escherichia coli*, and *Enterococcus cecorum* was also observed in IF birds. It is possible that the effect of the high flavonoid corn on the microbiota was overcome by the effect of NE, or that the positive effects of increased flavonoids in NE-challenged birds are a result of mechanisms which do not involve the microbiota. The effects of high flavonoid corn on NE infections may be further investigated as a possible alternative to antimicrobials.

## 1 Introduction

Poultry in particular broiler chickens continue to grow in popularity with consumers worldwide because they provide a nutritious and cost-effective source of food. Continued development of antimicrobial resistant bacteria has driven many countries worldwide to remove products which were previously used as growth enhancers and anti-bacterials. This has driven the poultry industry to seek novel alternatives to controlling diseases and enhancing growth (Kaldhusdal et al., 2016).

Necrotic enteritis (NE) in chickens is a disease caused by the Gram positive bacteria *Clostridium perfringens* (Timbermont et al., 2011) and has been historically controlled by addition of antimicrobials in diet (Mak et al., 2022). Clinical outbreaks of NE are characterized by inappetence, reduction in weight gain, ruffled feathers, diarrhea, lethargy, abrupt mortality, and the presence of characteristic lesions in the intestine (Timbermont et al., 2011). These bacteria are part of the normal microbiota and can result in NE in intensively produced broilers. The sole presence of *C. perfringens* does not always result in disease. There are predisposing factors to NE such as *Eimeria maxima* infection, feed with elevated content of non-starch polysaccharides, presence of mycotoxins, high stocking density, and immune status (Branton et al., 1987; Tsiouris et al., 2015).

It was previously reported that feeding broiler chickens a diet high in a flavonoid-rich corn variety (PennHFD1, a proprietary corn variety developed by Penn State University) ameliorated NE by reducing intestinal lesions and mortality. PennHFD1 also improved production parameters such as weight gain and feed conversion ratios (FCR), compared to chickens consuming standard commercial corn (Buiatte et al., 2022). Flavonoids are polyphenolic compounds produced by plants that have anti-inflammatory properties including scavenging and inhibiting the formation of reactive oxygen species (ROS) (Kumar and Pandey, 2013). In plants, flavonoids play an important role in protection against microbial infections (Kumar and Pandey, 2013). Flavonoids can inactivate bacterial adhesins and interfere with bacterial membranes, as well as interfere with enzymes that are involved in molecular transport and DNA and RNA synthesis. It has been previously reported that flavonoid compounds have an effect on *C. perfingens “in vitro”*, by elongating the cells and causing cellular damage *in vitro* (Noor-Mohammadi et al., 2019). Further studies determined that flavonoids derived from leaves of eucalyptus (*Eucalyptus globulus*) inhibited the growth of *C. perfringens* in experimentally infected broiler chickens (Ullah et al., 2021). These findings suggest that flavonoid compounds may have protective effects on chickens undergoing NE. Microbiota changes during *Eimeria* infection and NE have been documented by 16S sequencing and direct sampling, as well as PCR-based techniques. These changes can be characterized by increases in *E. coli* and *C. perfingens* (Daneshmand et al., 2022), decreases in butyrate producing bacteria (Stanley et al., 2012), and lactobacilli (Feng et al., 2010). However, it is apparent that results are often not consistent and are dependent on many factors, such as diet type, infection severity, age, breed, sex of the birds, and sampling time (Bindari and Gerber, 2022).

The goal of the current study was to determine the effect of the high flavonoid corn (PennHFD1) variety on the cecal microbiota of chickens undergoing clinical NE using 16s rRNA sequencing.

## 2 Materials and methods

### 2.1 Experimental design

The experimental design for this study as well as the performance parameters were previously reported (Buiatte et al., 2022), and it is also briefly summarized below. Four hundred, day-old straight run, broiler chickens (Ross 308 Aviagen) were randomly placed into 20 floor pens (2.6 m^2^). One hundred birds were used per experimental treatment with five replicates each (20 chicks per replicate). The basal diet used in the present study contained wheat and fishmeal which are factors known to induce NE in broiler chickens (Fathima et al., 2022). The exact diet composition and nutritional analysis are described in Buiatte et al., 2022, however **Tables S1**, **S2** which contain this information have also been included in the **Supplementary Materials**. The experiment was conducted for 21 days using a completely randomized design with four treatments groups: CTRL-A, non-infected birds eating commercial corn; CTRL-B, non-infected birds eating corn high in flavonoids (PennHFD1); IF-A, birds infected with *E. maxima* and *C. perfringens* eating commercial corn; and IF-B birds infected with *E. maxima* and *C. perfringens* eating PennHFD1 (Buiatte et al., 2022). Birds remained on their respective diets for the duration of the study and were housed using brooding conditions recommended by Aviagen. All procedures were approved by the Institutional Animal Care and Use Committee at The Pennsylvania State University (approval date 08/07/2020, protocol number PROTO202001566).

### 2.2 Infection model and bird sampling

On day 13 chicks were infected by oral gavage with 5,000 *E. maxima* oocysts (maintained at Beltsville Agricultural Research Center, in Beltsville, MD). The *E. maxima* APU2 strain was isolated in 2015 from oocysts obtained from a commercial chicken farm on the Eastern Shore of Maryland (Jenkins et al., 2017). This strain is propagated through chickens every three months, and purity is ensured by microscopic visualization and PCR. Aliquots of three strains of *C. perfringens*, two NetB positive and one NetB negative, isolated from field cases of NE, were inoculated into thioglycollate medium (Neogen^®^, Lansing, MI) and incubated anaerobically at 37°C for 24 hours to reach a concentration of 1 x 10^9^ CFU/mL. Anaerobiosis was achieved with a pouch of AnaeroPack^®^ System (Mitsubishi Gas Chemical America, New York, NY). Twelve hours before the first inoculation with *C. perfringens*, on day 17 feed from all pens was removed. Inoculation was done by top dressing the inoculum on top of the feed at a dose of 1 mL of 1 x 10^9^ CFU of *C. perfringens* per bird and returning the feed to the bird pens on day 18. Birds were inoculated again on day 19 via top dressing the inoculum onto the feed. On day 21, a total of 100 birds (five to eight birds/replicate) were randomly selected, euthanized by cervical dislocation, and necropsied for intestinal evaluation and sample collection (Buiatte et al., 2022). Samples of the cecal lumen (CE-L) and epithelial mucosa (CE-M) were taken for 16S rRNA sequencing. Isolated specimens were snap-frozen in liquid nitrogen and stored at -80°C until bacterial DNA isolation.

### 2.3 DNA isolation and library preparation

DNA was extracted from cecal scrapings and contents and was evaluated as described previously (Campos et al., 2022). The 16S rRNA gene amplicon libraries were generated using the workflow and chemistry supplied by Illumina (Illumina, Inc., San Diego, CA) and PCR primers (Forward: 5’-TCGTCGGCAGCGTCAGATGTGTATAAGAGACAGCCTACGGGNGGCWGC AG-3’ and Reverse: 5’-GTCTCGTGGGCTCGGAGATGTGTATAAGAGACAGGACTACHV GGGTATCTAATCC-3’) targeted the V3-V4 variable region of the 16S gene. Amplicon PCR followed by index PCR and PCR amplicon cleaning were performed as described previously (Campos et al., 2022). The concentration and quality of the amplicons were determined using QIAxcel DNA Hi-Resolution cartridge, proprietary QIAxcel ScreenGel software (version 1.6.0, www.qiagen.com), and QIAxcel Advanced System (Qiagen) per manufacturing instructions. The pooled (96 barcoded amplicons) DNA library (4 nM) and PhiX control v3 (Illumina, Inc., 4 nM) were denatured with 0.2 N NaOH (Sigma-Aldrich, Corp., St. Louis, MO) and diluted to a final concentration of 4 pM. The library was mixed with PhiX control (20% v/v) and pair-end 2 × 300-bp sequencing was performed using the Illumina MiSeq platform and a MiSeq Reagent Kit v3 (Illumina, Inc). The 16S rRNA gene sequences determined in this study were deposited in the NCBI Sequence Read Archive (SRA) database (SRA accession # PRJNA931944).

### 2.4 16S rRNA gene sequence, data processing and analysis

Quantitative Insight Into Microbial Ecology (QIIME) software package 2 (version 2021.4.0, http://qiime2.org), (Bolyen et al., 2019) was used to perform quality control and analysis of the sequence reads as described before (Campos et al., 2022). Raw fastq files were demultiplexed using q2-demux and quality filtered and dereplicated with q2-dada2 (Campos et al., 2022). Sequences with an average Phred score lower than 25 were removed. Representative sequence sets for each dada2 sequence variant were used for taxonomy classification. MAFFT was used for multiple sequence alignment and Fastree was used to generate phylogenetic trees. Naïve Bayesian classifier was used for taxonomic classification against the Greengenes database v. 13_8 (http://greengenes.secongenome.com) (Proszkowiec-Weglarz et al., 2020). Data were rarefied to the lowest possible counts of sequences per sample for calculation of alpha and beta diversities. Alpha diversity indices [Amplicon Sequence Variants (ASVs), Shannon’s diversity index, Pielou’s Evenness (evenness), and Faith’s Phylogenetic Diversity (richness)] were obtained through QIIME 2 package. Analysis of beta diversity was performed by QIIME2 employing Unweighted UniFrac. Principal coordinate analysis (PCoA) based on Unweighted UniFrac distance metrics was implemented in the QIIME2 software. Linear Discriminant Analysis (LDA) Effect Size (LEfSe) algorithm was used to identify taxa with significant differential abundance between CTRL and IF birds (Segata et al., 2011).

### 2.5 Statistical analyses

Differences between alpha diversity indices were tested using the Kruskal–Wallis test (QIIME2). The difference in community structure due to main effects (infection status and feed type) and their interaction were statistically tested by non-parametric multivariate ANOVA (PERMANOVA) with 999 permutations using QIIME2 software package. Microbiota composition data were obtained by normalization to total number of reads in each sample (relative abundance) and were analyzed using two-way ANOVA using GLM (Statistical Analysis System (SAS) Software, Version 9.4, SAS Institute Inc, Cary, NC). Statistically significant differences were declared when p ≤ 0.05. For the LEfSe analysis, the alpha value was set at 0.5 and for the Kruskal–Wallis test the threshold for the log10LDA score was set at 2.0.

## 3 Results

The performance data from this study was previously published (Buiatte et al., 2022). This data represents further analysis of the previously obtained data.

### 3.1 Sequencing summary

A total of 10,215,430 reads were obtained from 23 CE-L samples with 444,149 average reads per sample. After trimming and quality control 3,558,078 reads remained, with 154,699 average reads per sample. The sequencing depth of CE-L samples used for analysis was 22,324. A total of 10,215,430 reads were obtained from 23 CE-M samples with 343,643 average reads per sample. After trimming and quality control 950,668 reads remained, with 41,333 average reads per sample. The sequencing depth of CE-M samples used for analysis was 10,680.

### 3.2 Alpha diversity

**Figure 1:**
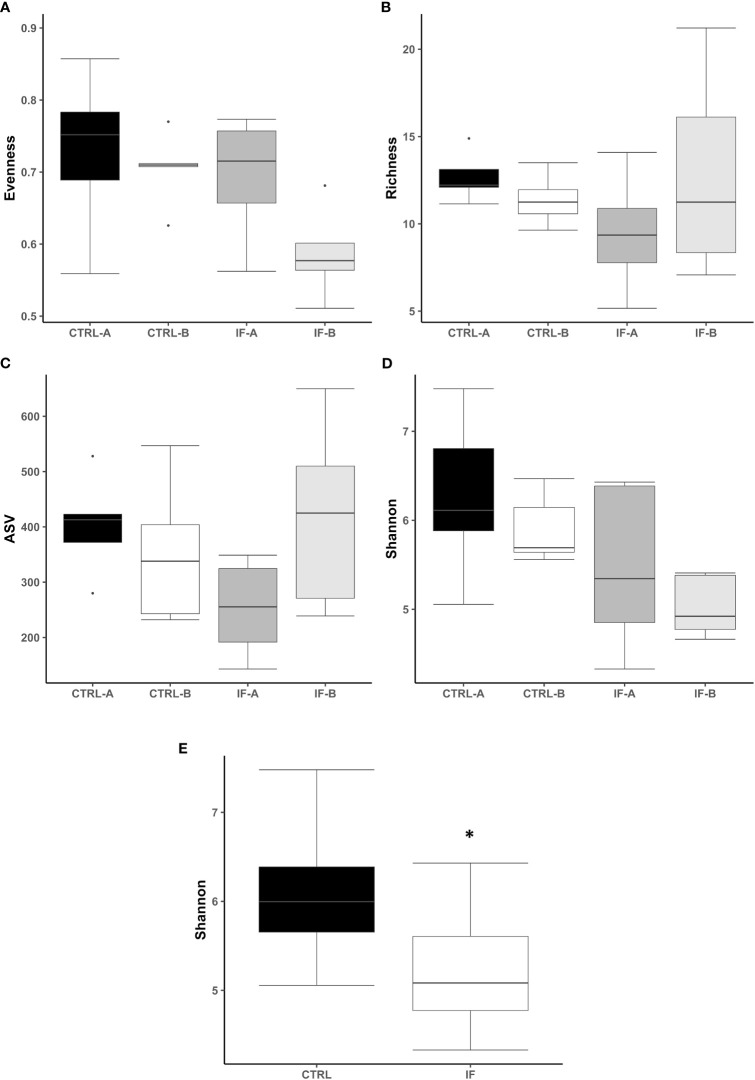
Effect of *Eimeria maxima/Clostridium perfringens* infection and diet type in the cecal lumen (CE-L) on alpha diversity indices **(A)** Evenness, **(B)** Richness, **(C)** number of amplicon sequence variants (ASV), and **(D)** Shannon index in cecal luminal bacterial population at 7 days post-infection. **(E)** Significant effect of infection on Shannon index. CTRL-A – uninfected animals consuming commercial corn, CTRL-B – uninfected animals consuming high flavonoid corn (PennHFD1), IF-A – infected animals consuming commercial corn, and IF-B (infected animals consuming high flavonoid corn (PennHFD1). Significant (*p* < 0.05) differences are indicated by an asterisk.

The effect of feed type (commercial corn versus high-flavonoid corn) and infection status (uninfected versus coccidia and *C. perfringens* coinfection) on alpha diversity in the CE-L is shown in **Table 1** and **Figure 1**. There were no significant differences on evenness, richness, ASV and Shannon diversity index between the four treatment groups (CTRL-A, CTRL-B, IF-A, and IF-B, **Figures 1A–D**). The Shannon diversity index was significantly different (*p =* 0.015) between samples from the infected and the uninfected samples, with IF samples having lower diversity than CTRL samples (**Figure 1E**).

**Table 1:** Kruskal–Wallis *p*-values for interactive Feed × IF (corn diet type x infection status) or main (Feed and IF) effects for alpha diversity indices in cecum lumen and mucosa.

	IF	Feed	Feed x IF
Lumen			
Evenness	0.153	0.120	0.099
Richness	0.080	0.576	0.130
ASV	0.170	0.260	0.070
Shannon	**0.015**	0.420	0.061
Mucosa			
Evenness	0.709	0.070	0.180
Richness	0.005	0.062	**0.009**
ASV	0.016	0.016	**0.011**
Shannon	0.070	0.709	0.310

ASV, amplicon sequence variant.

Values in bold indicate statistical significance (*p* < 0.05).

**Figure 2:**
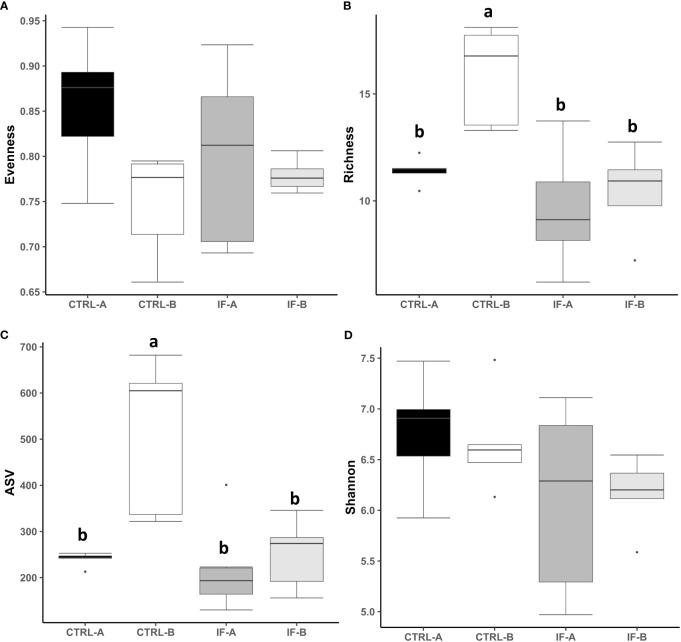
Effect of *Eimeria maxima/Clostridium perfringens* infection and diet type in the cecal mucosa (CE-M) on alpha diversity indices **(A)** Evenness, **(B)** Richness, **(C)** number of amplicon sequence variants (ASV), and **(D)** Shannon index in cecal mucosal bacterial population at 7 days post-infection. CTRL-A – uninfected animals consuming commercial corn, CTRL-B – uninfected animals consuming high flavonoid corn (PennHFD1), IF-A – infected animals consuming commercial corn, and IF-B (infected animals consuming high flavonoid corn (PennHFD1). ^a,b^ *p* < 0.05.

The effect of feed type (commercial corn versus high-flavonoid corn) and infection status (uninfected versus coccidia and *C. perfringens* coinfection) on alpha diversity in the CE-M is shown in **Table 1** and **Figure 2**. There were no significant differences in evenness and Shannon diversity index between any treatment groups (**Figures 2A, D**). Richness and ASV numbers were significantly (P<0.05) higher in uninfected chickens fed PennHFD1 (CTRL-B) in comparison to other groups (**Figures 2B, C**).

### 3.3 Beta diversity

**Figure 3:**
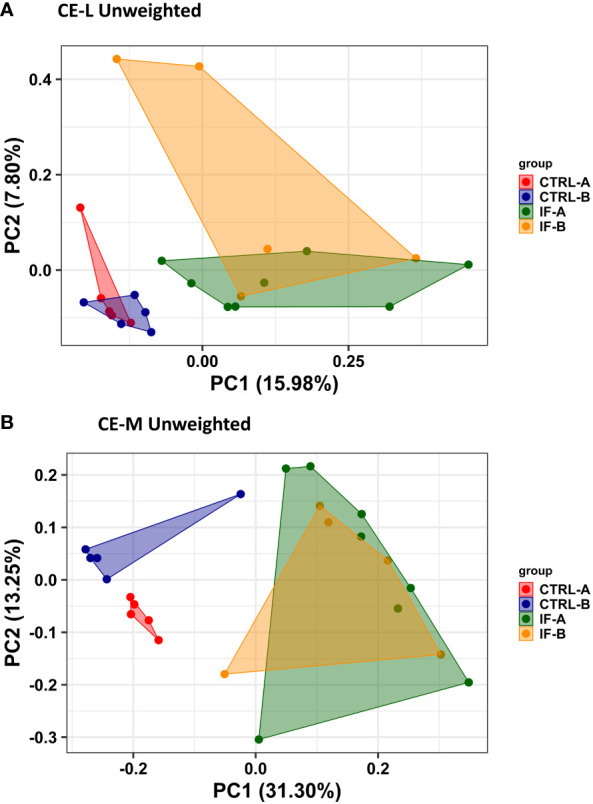
Effect of *Eimeria maxima/Clostridium perfingens* infection and diet type on beta diversity of cecal luminal **(A)** (CE-L), and cecal mucosa **(B)** (CE-M), bacterial populations using the Principal Coordinate analysis (PcoA) based on the Unweighted UniFrac distances between groups. CTRL-A – uninfected animals consuming commercial corn, CTRL-B – uninfected animals consuming high flavonoid corn (PennHFD1), IF-A – infected animals consuming commercial corn, and IF-B (infected animals consuming high flavonoid corn (PennHFD1).

PERMANOVA analysis was carried out based on the Unweighted UniFrac distance matrices and was used to determine the similarities between microbial communities residing in CE-L and CE-M. For PERMANOVA based on Unweighted Uni-Frac analysis there was significant (*p =* 0.001) interaction between the two main effects (IF x Feed) in both CE-L and CE-M. The beta diversity in CE-L calculated by Unweighted Uni-Frac analysis showed that bacterial communities from infected and uninfected birds were separated into distinct groups (IF versus CTRL) regardless of corn type (commercial or PennHFD1) (**Figure 3A**). In CE-M, bacterial communities from infected and uninfected birds (IF versus CTRL) also separated into distinct groups but additionally bacterial communities from uninfected birds were distinct depending on diet type, commercial (CTRL-A) or PennHFD1 (CTRL-B) (**Figure 3B**). In the Unweighted Uni-Frac analysis samples from CE-L and CE-M of IF animals showed higher variability compared to those of uninfected (CTRL) birds (**Figures 3A, B**).

### 3.4 Taxonomic Composition

**Figure 4:**
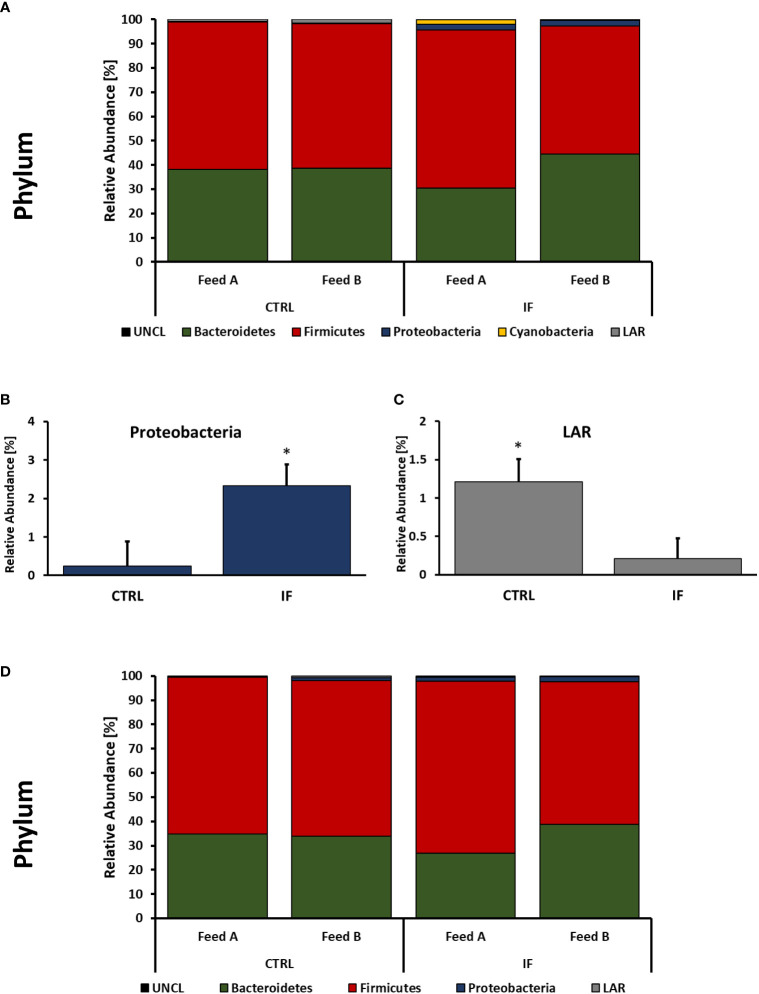
Effect of *Eimeria maxima/Clostridium perfingens* infection and diet type on relative bacterial abundance (%) in cecal lumen **(A–C)** and cecal mucosa **(D)** at phylum level. Statistically significant (*p* < 0.05) differences in Proteobacteria **(B)** and Low Abundance Reads (LAR) **(C)** between uninfected (CTRL) and infected (INF) are indicated by an asterisk.

Taxonomic composition of bacterial communities at the phylum level in CE-L and CE-M from all four treatment groups is presented in **Figure 4**. Also, representative sequences, taxonomic assignment, and frequency of reads from CE-M and CE-L have been provided in **Supplementary Tables 3**, **4**, respectively. Members of six putative phyla were present in the CE-L: Unclassified bacteria (UNCL), Bacteroidetes, Firmicutes, Proteobacteria, Cyanobacteria, and Low abundance reads (LAR). Bacteroidetes and Firmicutes were the most common phyla in both CE-L and CE-M. The levels of Proteobacteria were significantly (*p =* 0.0239) higher in infected birds (**Figure 4B**) while LAR were significantly lower (*p =* 0.0216) in infected birds (**Figure 4C**). The taxonomic composition of CE-M at the phylum level was similar (*p* > 0.05) between all treatment groups (**Figure 4D**).

**Figure 5:**
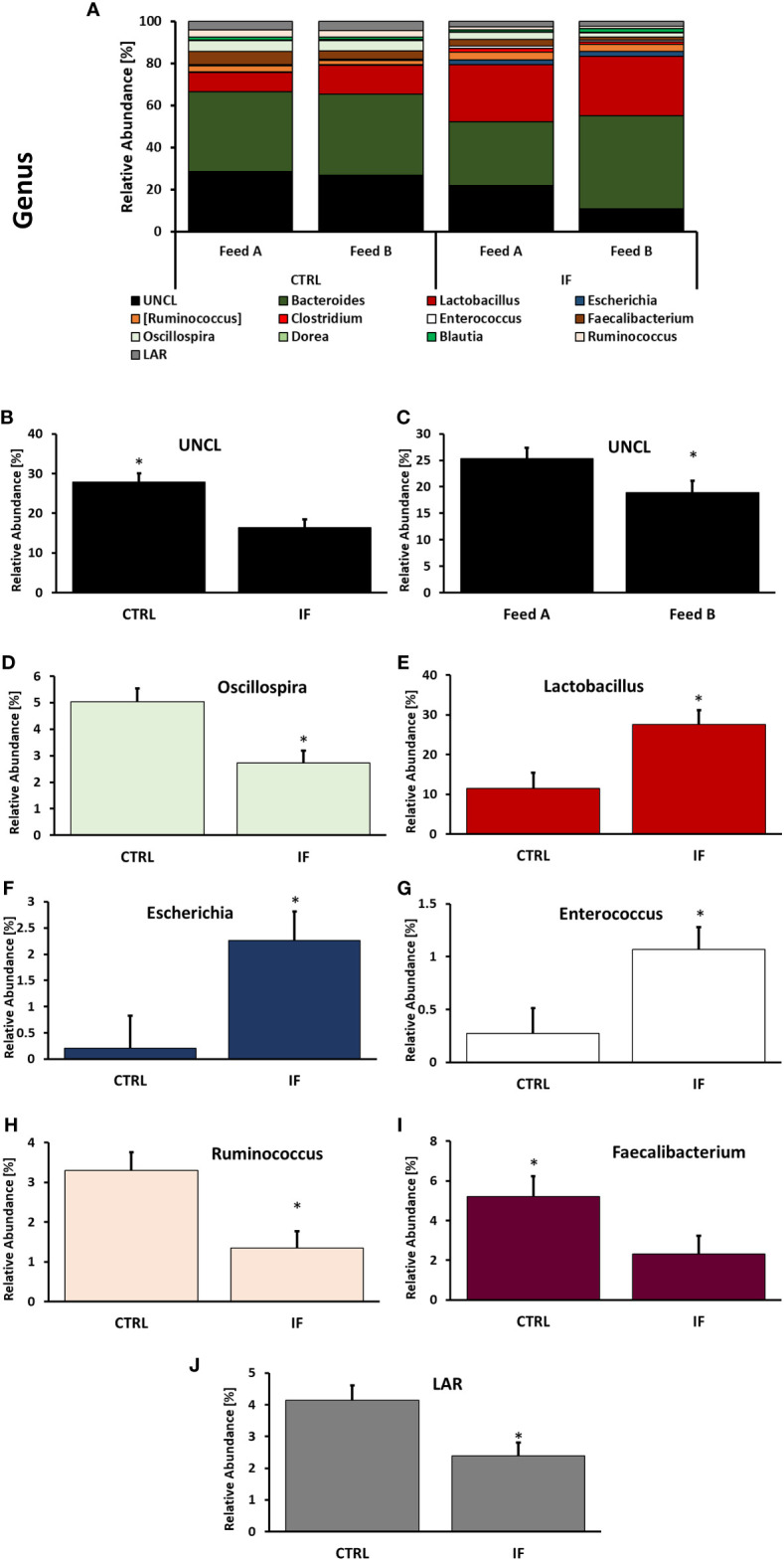
Effect of *Eimeria maxima/Clostridium perfingens* infection and diet type on relative bacterial abundance (%) in cecal lumen **(A–J)** at genus level. Statistically significant (*p* < 0.05) differences in **(B)** UNCL, **(D)** *Oscillospira*, **(E)** *Lactobacillus*, **(F)** *Escherichia*, **(G)** *Enterococcus*, **(H)** *Ruminococcus* (members of family Ruminococcaceae), **(I)** *Faecalibacterium*, **(J)** LAR bacteria between uninfected (CTRL) and infected (INF) birds; and **(C)** UNCL bacteria between diet type (Feed A and Feed B) are indicated by an asterisk.

**Figure 6:**
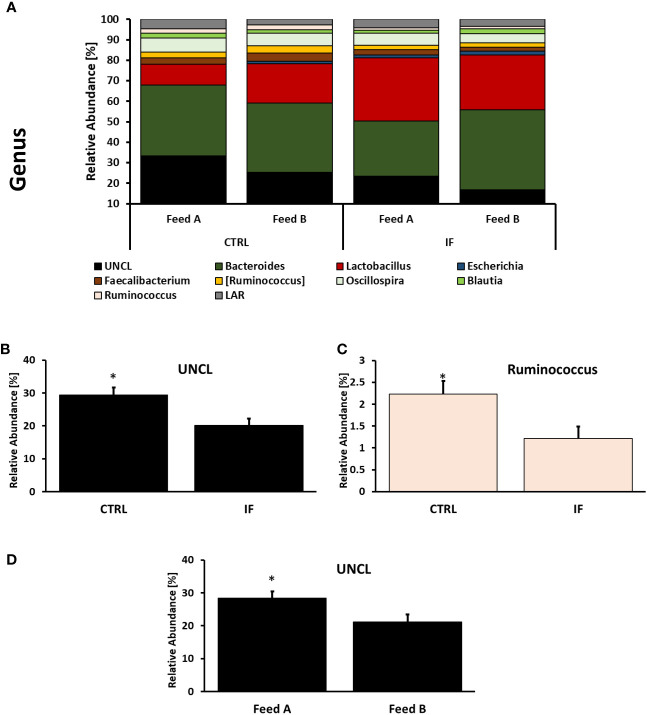
Effect of *Eimeria maxima/Clostridium perfingens* infection and diet type on relative bacterial abundance (%) in cecal mucosa **(A–D)** at genus level. Statistically significant (*p* < 0.05) differences in **(B)** UNCL bacteria, **(C)** *Ruminococcus* (members of family Ruminococcaceae, [Ruminococcus] refers to members of family Lachnospiraceae), between uninfected (CTRL) and infected (INF) birds; and **(D)** UNCL bacteria between diet type (Feed A and Feed B) are indicated by an asterisk.

Taxonomic composition of bacterial communities at the genus level in CE-L and CE-M, from all four treatment groups is presented in **Figures 5**, **6**, respectively. Among the genera identified in CE-L (**Figure 5A**), UNCL, *Bacteroides*, and *Lactobacillus* were the most abundant. Relative abundance of UNCL bacteria was significantly affected by infection (**Figure 5B**) as well as corn type (**Figure 5C**). Infected birds had lower relative abundance of UNCL in comparison to CTRL birds (**Figure 5B**) while the birds fed PennHFD1 were characterized by lower levels of UNCL (**Figure 5C**). Relative abundance of *Oscillopira* (**Figure 5D**), (*p =* 0.0037), *Ruminoccoccus* (member of Ruminococcaceae) (**Figure 5H**), (*p =* 0.0053), *Faecalibacterium* (**Figure 5I**), (*p =* 0.0479), and LAR (**Figure 5J**), (*p =* 0.0029) were significantly lower in IF birds. On the other hand, relative abundance of *Escherichia* (**Figure 5F**), (*p =* 0.0233), *Lactobacillus* (**Figure 5E**), (*p =* 0.0071), and *Enterococcus* (**Figure 5G**), (*p =* 0.0244) were significantly higher in IF birds in comparison to CTRL birds. Among genera identified in CE-M (**Figure 6A**), UNCL, *Bacteroides*, and *Lactobacillus* were the most abundant. Levels of UNCL bacteria were significantly affected by infection (**Figure 6B**) and feed type (**Figure 6D**), with relative abundance being lower in infected birds (*p =* 0.083) and in birds consuming PennHFD1 (*p =* 0.031). The relative abundance of *Ruminococcus* (member of Ruminococcaceae) was significantly lower in IF birds (**Figure 6C**), (*p =* 0.0259).

**Figure 7:**
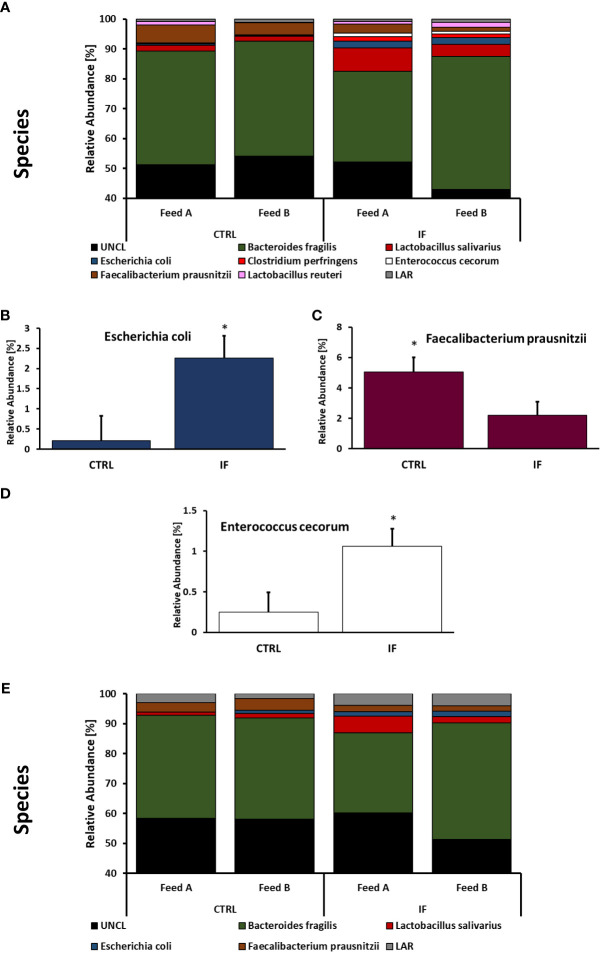
Effect of *Eimeria maxima/Clostridium perfingens* infection and diet type on relative bacterial abundance (%) in cecal lumen **(A–D)** and cecal mucosa **(E)** at species level. Statistically significant (*p* < 0.05) differences in **(B)** *Escherichia coli*, **(C)** *Faecalibacterium prausnitzii*, and **(D)** *Enterococcus cecorum* between uninfected (CTRL) and infected (INF) birds are indicated by an asterisk.

Taxonomic composition of bacterial communities at the species level in CE-L and CE-M from all four treatment groups is presented in **Figure 7**. Among species identified from CE-L (**Figure 7A**), UNCL and *Bacteroides fragilis* were the most abundant. Relative abundance of *Escherichia coli* (**Figure 7B**), (*p =* 0.0233), and *Enterobacter cecorum* (**Figure 7D**), (*p =* 0.021), was significantly higher in IF birds while *Faecalibacterium prausnitzii* (**Figure 7C**), (*p =* 0.0458), was significantly lower in IF birds. Among species identified in CE-M (**Figure 7E**), UNCL and *Bacteroides fragilis* were the most abundant. There were no significant differences among treatment groups at the species level in CE-M (**Figure 7E**).

**Figure 8:**
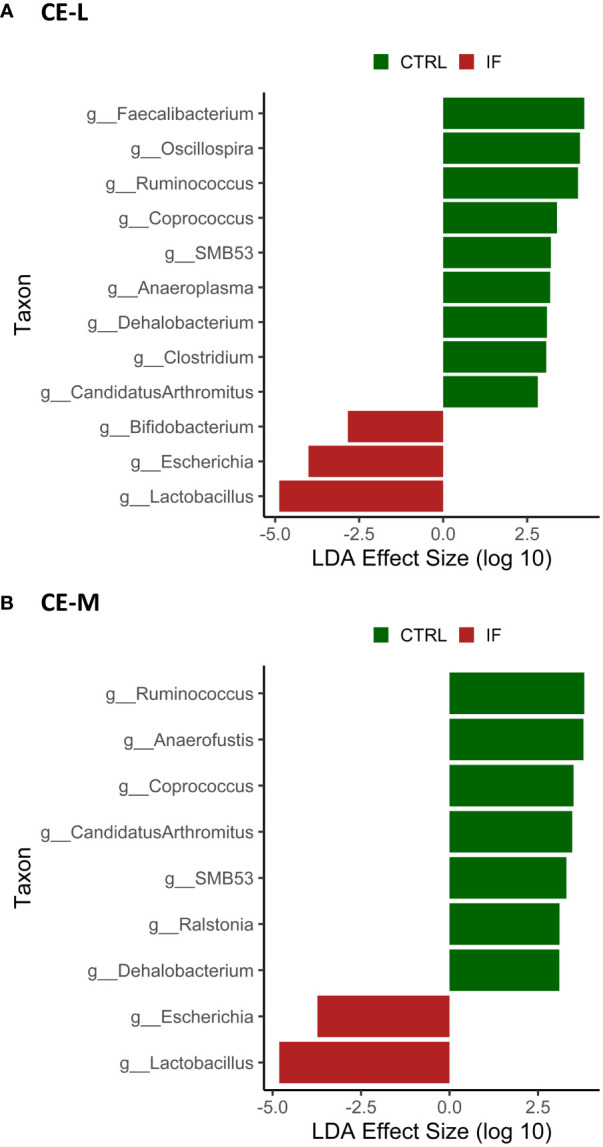
Effect of *E. maxima/C. perfringens* on differentially abundant bacterial genera as determined by Linear Discriminant Analysis (LDA) effect size (LEfSe) analysis in cecal lumen (CE-L, **(A)**) and mucosal (CE-M, **(B)**) bacterial populations. CTRL- uninfected chickens, IF- infected chickens. Genus *Ruminococcus* refers to members of family Ruminococcaceae.

LEfSe analysis was performed to determine differentially abundant genera in both CE-L and CE-M. The LefSe analysis found significant differences in abundance between CTRL and IF birds in both CE-L and CE-M and the results are shown in **Figures 8A, B**, respectively. No significant differences in bacterial abundance were found between birds eating commercial corn versus PennHFD1. At the genus level, *Lactobacillus*, *Escherichia*, and *Bifidobacterium*, were more abundant in CE-L of IF birds. *Candidatus arthromitus*, *Clostridium*, *Dehalobacterium*, *Anaeroplasma*, SMB53, *Coprococcus*, *Ruminococcus* (member of Ruminococcaceae), *Oscillospira*, and *Faecalibacterium* were more abundant in CE-L of CTRL birds. At the genus level of CE-M *Lactobacillus* and *Escherichia* were more abundant in IF birds, and *Dehalobacterium*, *Ralstonia*, SMB53, *Candidatus arthromitus*, *Coprococcus*, *Anaerofustis*, and *Ruminococcum* were more abundant in CTRL birds.

## 4 Discussion

In this manuscript, we report results of the analysis of the cecal microbiota (both luminal as well as mucosal scrapings) in broiler chickens with clinical NE fed a diet containing commercial corn or corn high in flavonoids (PennHFD1). Our previous study (Buiatte et al., 2022) demonstrated that NE affected chicks that were fed PennHFD1 had decreased incidence of intestinal lesions (from 55.5% to 29.6%) as well as a reduction in mortality by 42.93%. In addition, the chickens eating PennHFD1 had lower FCR, and higher weight gain compared to chickens consuming commercial corn (in both IF and CTRL animals). Because of improved performance parameters further analysis of the cecal microbiota was carried out, to determine if the high flavonoid corn influenced the microbiota, and whether it mitigated any changes in the microbiota caused by NE.

### 4.1 Effect of infection and diet type on alpha diversity

Necrotic enteritis is caused by *C. perfringens* in chickens with predisposing factors such as diet and infection with *Eimeria*. In the current study, wheat, fishmeal, and infection with *E. maxima* were used to predispose the chickens to NE. It has been previously reported (Feng et al., 2010) that a mixed species *Eimeria* infection with or without *C. perfringens* decreased ileal microbiota alpha diversity of the digesta. Analysis of cecal contents of birds with subclinical NE also showed a decrease in alpha diversity (Yu et al., 2022). It is important to note that microbial diversity (both alpha and beta) can be influenced by many factors, such as housing type (new or used litter, floor pen or battery cage) or vaccination status against coccidia (Bortoluzzi et al., 2019). Our data showed that alpha diversity (ASV and Richness) in CTRL-B group was significantly higher than in the other three groups, suggesting that addition of corn high in flavonoids had a positive effect on the diversity of the microbiota. It is known that a diverse microbiota is correlated with good gut health (Lozupone et al., 2012) and flavonoid compounds may improve its diversity.

### 4.2 Effect of infection and diet type on beta diversity

Unweighted Uni-Frac analysis was carried out to determine beta diversity in CE-M and CE-L. Unweighted Uni-Frac showed that samples from IF chickens were distinct from those of CTRL birds, therefore, microbial populations from infected animals are more similar to each other, than to those of uninfected samples. Differences in beta diversity in the ceca have also been previously reported in birds infected with *Eimeria* alone (Campos et al., 2022). Stanley et al. (2014) reported that infection with *C. perfringens* alone did not have a significant effect on beta diversity, however, when birds were infected with a combination of *Eimeria*, *C. perfringens*, and were fed a fishmeal diet statistical differences in beta diversity of cecal microbiota were observed. The effect of high-flavonoid corn on species abundance was only observed in Uni-Frac analysis of CE-M in CTRL birds, where taxa from CTRL-B (high flavonoid corn) birds formed a distinct group. In the remainder of the Uni-Frac analysis, the impact of infection might have over-ridden any effect of high flavonoid corn on bacterial taxa present in the CE-M.

### 4.3 Effects of infection and diet on taxonomic composition of the microbiota

Taxonomic composition of the microbiota at the phylum, genus, and species level was analyzed in both CE-L and CE-M. Most of the significant differences were due to infection. The effect of diet only resulted in significantly higher abundance of unclassified bacteria at the genus and species level in CTRL-A birds. At the phylum level Proteobacteria were more abundant in CE-L of IF animals while LAR bacteria were more abundant in CTRL animals. The phylum Proteobacteria contains over 460 genera many of which can be pathogenic such as *Escherichia*, *Campylobacter*, and *Salmonella.* Members of phyla Proteobacteria and Firmicutes are typically the most abundant in the ceca, and an increase in Proteobacteria has been documented in *E. maxima* infected birds (Jebessa et al., 2022). An increase in Proteobacteria has also been observed in the ileum of NE infected birds (Xu et al., 2018). An increase in Proteobacteria has also been noted in other species with gastrointestinal disturbances (Sun et al., 2019), and is therefore, not specific to NE or coccidiosis, but could be considered a hallmark of dysbiosis. The phylum Firmicutes contains over 250 genera, including Clostridia and Bacilli. Changes in the population of Firmicutes have been observed in the ileum of NE infected birds (Xu et al., 2018) however, no differences were observed in our study.

Most of the significant taxonomic changes were observed at the genus level, with differences between 8 genera between CTRL and IF birds in the CE-L. The effect of diet was only noted in the increase of UNCL bacteria in CE-M and CE-L of birds eating the commercial corn diet (CRTL-A). In the CE-M only two genera significantly differed between CTRL and IF birds, therefore, genera in the cecal lumen appear to be more affected by NE. The effect of infection resulted in the decrease of three genera (*Oscillospira*, *Ruminococcus*, *Faecalibacterium)* and one species (*Faecalibacterium prausnitzii*) in CE-L which are known or are hypothesized to produce short chain fatty acids (SCFA) (Gophna et al., 2017; Krga and Glibetic, 2022). *Ruminococcus* was also decreased in CE-M. The genus *Ruminococcus* refers to members of family Ruminococcaceae which tend to include beneficial bacteria. Short chain fatty acids (SCFA) are produced by bacteria through fermentation of carbohydrates that cannot be digested by the host and can be used by bacteria as a source of energy. However, SCFA are also beneficial to the host and can contribute up to 10% of the total human caloric intake (den Besten et al., 2013). Maintenance of the intestinal barrier by regulation of tight junction proteins, stimulation of mucin production, prevention of translocation of pathogenic bacteria (Krga and Glibetic, 2022) and increase in the secretion of antimicrobial peptides and various other immune regulators (Corrêa-Oliveira et al., 2016) can be influenced by SCFAs. Interestingly, Latorre et al., 2018 found increased levels of *Ruminococcus* and *Oscillospira* in the ileal contents, and Xu et al., 2018 also found increased levels of *Ruminococcus* in chickens challenged with NE, therefore, variation in findings among studies is often present. Variability in microbiota studies is often encountered, possibly due to sampling numbers, differences in experimental conditions, and differences in individual experimental subjects.

We found that genus *Lactobacillus* was increased in IF animals. This genus includes lactic acid producing bacteria and is thought to be a beneficial component of the microbiota. This finding was surprising since often *Lactobacilli* tend to decrease in association with NE (Antonissen et al., 2016), however, Latorre et al., 2018 reported increased *Lactobacillus* in the ileal contents of chickens with NE. Lin et al., 2017 also reported that *Lactobacillus* increased in cecal contents of birds with NE. The increase in *Lactobacillus* during NE could be a protective response by the gut, however, the role that lactic acid bacteria play during infections needs to be further investigated.

*Enterococcus* and *Escherichia* are present in the normal microflora of the gut (Krga and Glibetic, 2022; Mak et al., 2022), however, members of these genera can be perturbed during infections. We found that both genera were increased in the CE-L of chickens with NE. Specifically, *Escherichia coli* and *Enterococcus cecorum* were significantly elevated in NE infected birds. *E. coli* in poultry is known to have many strains that are resistant to antimicrobials and can be a cause of gastroenteritis in humans as well as animals (Bonnet et al., 2009), therefore, it is an opportunistic pathogen with zoonotic capabilities. Pathogenic *E. cecorum* in chickens causes a condition called “kinky-back” where a mass caused by inflammation develops around free thoracic vertebrae resulting in paralysis. Predisposition to “kinky-back” is thought to be linked to leakiness of the gut caused by infections or intestinal inflammation (Jung et al., 2018). Presence of *E. cecorum* has been previously found in the ileal contents of chickens with severe NE lesions (Yang et al., 2021), therefore, NE could contribute to incidence of “kinky-back”.

For this study we collected both the luminal contents of the ceca as well as mucosal scrapings to include the bacteria which are attached to the intestinal epithelium. Most of the taxonomic differences were noted in the CE-L, however, it is important to include the mucosal populations in the analysis since they are made up of different species with different functions compared to the luminal bacteria.

## 4.4 Conclusions

In our study the effect of diet (commercial vs. high flavonoid corn) was much smaller compared to the effect of infection. It is possible that the feeding of corn rich in flavonoids does not alter the microbiota, and that infection with *E. maxima* and *C. perfringens* results in microbiota changes that are much greater than those of flavonoid compounds. The addition of high flavonoid corn improved the symptoms and effects of NE, but the mechanisms behind these effects did not center primarily on the microbiota.

## Data availability statement

The datasets presented in this study can be found in online repositories. The names of the repository/repositories and accession number(s) can be found below: https://www.ncbi.nlm.nih.gov/, PRJNA931944.

## Ethics statement

The animal study was approved by Beltsville Agricultural Research Center IACUC. The study was conducted in accordance with the local legislation and institutional requirements.

## Author contributions

KM, AL, VB, and MP-W contributed to the conception, design, and investigation of the study. MP-W conducted the bioinformatics analysis. MJ provided *Eimeria* used in infection and MM, SC, and TL, provided the PennHFD1 corn. KM wrote the first draft of the manuscript. KM, VB, AL, MM, TL, SC, MJ, and MP-W contributed to manuscript revision. All authors contributed to the article and approved the submitted version.
