# Supplementary material for: Comparative analysis of microbiota in the ceca of broiler chickens with necrotic enteritis fed a commercial corn diet or with corn high in flavonoids (PennHFD1)
Source: Front Microbiomes. 2023 Oct 25;2:1212130. doi: 10.3389/frmbi.2023.1212130 (PMC12993490; doi:10.3389/frmbi.2023.1212130)
Supplement: Supplementary file 1 [file DataSheet_1.zip › Supplementary Tables 1-2.DOCX]

**Supplementary Table 1.** Composition of the diet treatments (Feed A and Feed B) fed to broiler chickens in an experiment of necrotic enteritis.

| **Item** | **Composition (%)** |
| --- | --- |
| Corn* | 31.53 |
| Wheat | 30.00 |
| Soybean meal | 20.20 |
| Fish meal | 15.00 |
| Lard | 2.57 |
| Vitamin and Mineral premix | 0.40 |
| Salt | 0.30 |

*The source of corn varied in the two diets formulated for the experiment. Feed A (commercial corn); Feed B (high-flavonoid corn, PennHFD).

**Supplementary Table 2**. Nutrient composition analysis of the two used sources of corn.

| **Composition** | **Commercial corn** | **PennHFD** |
| --- | --- | --- |
| Moisture | 8.8 | 9.1 |
| Dry Matter | 91.2 | 90.9 |
| Crude Protein | 13.2 | 12.9 |
| Lignin (%DM) | 2.02 | 2.23 |
| Crude fat (%DM) | 4.03 | 4.29 |
| Ash (%DM) | 1.85 | 2.07 |
| Calcium (%DM) | 0.01 | 0.02 |
| Phosphorus (%DM) | 0.33 | 0.40 |
| Magnesium (%DM) | 0.11 | 0.15 |
| Potassium (%DM) | 0.40 | 0.35 |
| Sodium (%DM) | 0.01 | 0.01 |
| Iron (PPM) | 31 | 95 |
| Manganese (PPM) | 10 | 17 |
| Zinc (PPM) | 26 | 27 |
| Copper (PPM) | 2 | 4 |
| ME (Mcal/lb) | 1.52 | 1.49 |

PennHFD: flavonoid-rich corn cultivar developed at The Pennsylvania State University
